# Fabrication of transparent hemispherical 3D nanofibrous scaffolds with radially aligned patterns via a novel electrospinning method

**DOI:** 10.1038/s41598-018-21618-0

**Published:** 2018-02-21

**Authors:** Jeong In Kim, Ju Yeon Kim, Chan Hee Park

**Affiliations:** 1 Department of Bionanosystem Engineering, Graduate School, Chonbuk National University, Jeonju, 561–756 Republic of Korea; 2 Division of Mechanical Design Engineering, College of Engineering, Chonbuk National University, Jeonju, 561-756 Republic of Korea

## Abstract

Tissue engineering has significantly contributed to the development of optimal treatments for individual injury sites based on their unique functional and histologic properties. Human organs and tissue have three-dimensional (3D) morphologies; for example, the morphology of the eye is a spherical shape. However, most conventional electrospinning equipment is only capable of fabricating a two-dimensional (2D) structured fibrous scaffold and no report is available on a 3D electrospinning method to fabricate a hemispherical scaffold to mimic the native properties of the cornea, including microscopic to macroscopic morphology and transparency. We proposed a novel electrospinning method using a single nonconductive hemispherical device and a metal pin. A designed peg-top shaped collector, a hemispherical nonconductive device with a metal pin in the center and copper wire forming a circle around at the edge was attached to a conventional conductive collector. A 3D hemispherical transparent scaffold with radially aligned nanofibers was successfully fabricated with the designed peg-top collector. In summary, our fabricated 3D electrospun scaffold is expected to be suitable for the treatment of injuries of ocular tissues owing to the hemispherical shape and radially aligned nanofibers which can guide the direction of the main collagen and cellular actin filament in the extracellular matrix.

## Introduction

Recently, in the area of tissue engineering, research has focused on developing scaffolds that can repair or replace the functions of damaged tissues or organs. A scaffold mimicking an extra cellular matrix (ECM) designed by tissue engineering is used for wound sites^1,2^. The ideal scaffold mimics the ECM structure of the target primary tissue of the target tissue^3^. Since the ECM plays an important role in tissues and determines the survival or functional maintenance of cells, studies have been carried out to imitate the cytoplasmic matrix^4,5^. Supports such as a nerve conduit, an eardrum, and a cornea have been constructed by surface patterning using electrospinning to induce cell alignment^6,7^. Electrospinning has played an important role in the development of nanofibrous scaffolds for clinical use such as in tissue engineering. Polymer solutions or polymers are forced through an electric field which then elongates the polymer droplet, resulting in the formation of uniform fibers with nano to micrometer-scale diameters.

In particular, biomaterials for corneal tissue engineering must demonstrate several important functions for their potential utility *in vivo*, including transparency, biocompatibility and slow biodegradability^8–11^. A typical treatment for injured corneal tissue is an implantation of a patch of amniotic membrane in which the cornea cells or stem cells are cultured^12^. However, since it is difficult to obtain a human amniotic membrane (hAM) for therapeutic use, attention has been paid to developing an alternative carrier having the immune-privileged, anti-inflammatory, and growth-promoting properties of amniotic membrane for the ocular surface reconstruction. Various polymeric materials have attracted interest as an alternative to this biological amniotic membrane due to their ability to meet specific needs while being able to be mass produced.

In the field of materials science, various applicable biomaterials have been identified, ranging from synthetic polymers to natural polymers, which are highly soluble, inexpensive, easy to process, and biocompatible. While native polymers show better cell attachment and compatibility, they have poor mechanical properties. Therefore, functionalized electrospun fibers with improved properties need to be fabricated in combination with synthetic polymers. Among these electrospun fibers, polycaprolactone (PCL) is thermoplastic polyester with hydrophobic and semi-crystalline properties, which has been approved by the FDA for human medical applications^13,14^. Because of the advantageous properties of PCL, it has been researched in a number of studies involving mixtures with various other polymers.

Natural materials, collagen which has excellent biocompatibility and biodegradability have been extensively utilized for the manufacturing of corneal scaffold^15^. The human cornea with a thickness of about 500 µm is composed of the stroma with its keratocytes and aligned collagen fibers^16^. Connon *et al*. have confirmed that ordered collagen fibrils and fibers shown to be denser, thicker, and having a higher mechanical strength than that of randomly oriented constituents^17^. Also, Lindsay *et al*. proposed an electrospun scaffold containing collagen I, which replicated the unique arrangement, alignment, and morphological cues of the fibers of collagen I in the native corneal tissue^9^.

**Figure 1:**
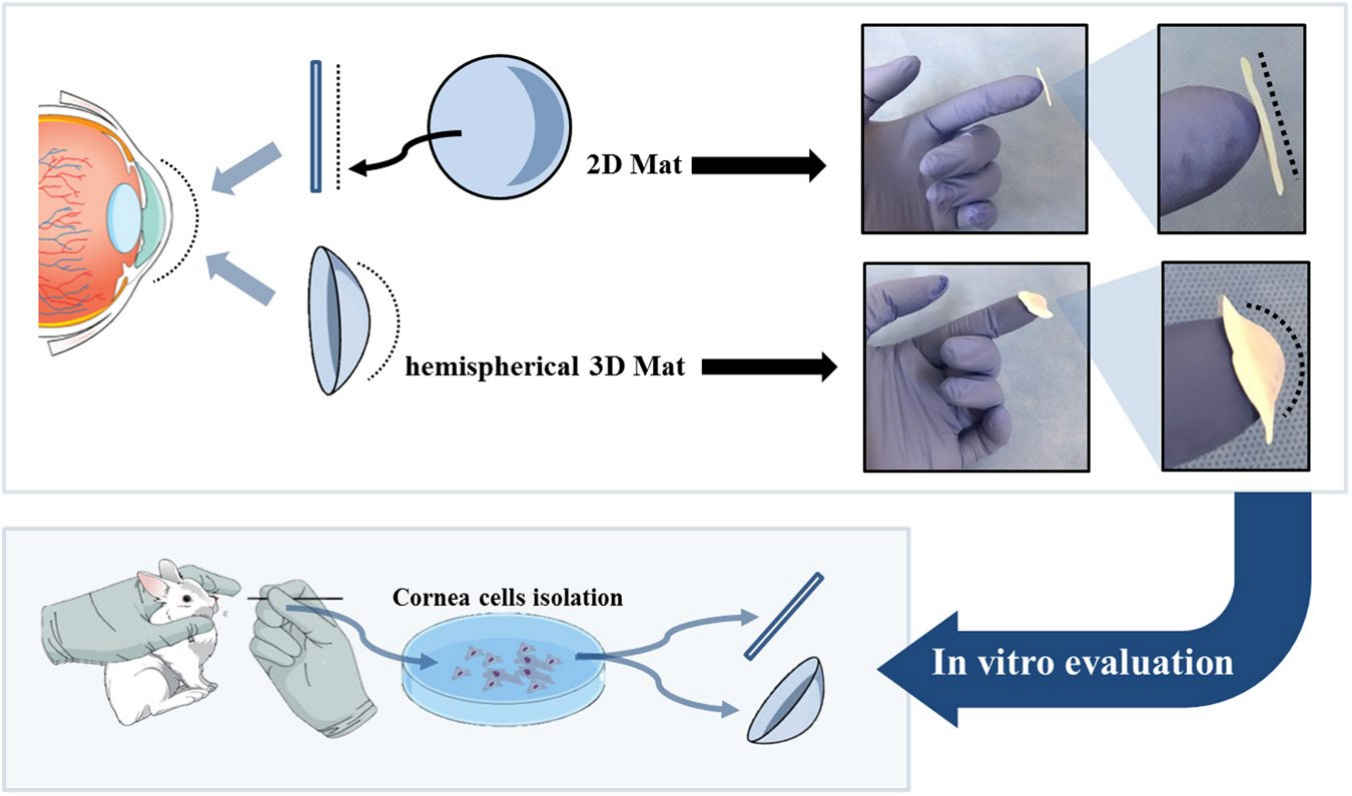
Illustration of the shape comparison of 3D and 2D nanofibrous scaffolds and experimental process.

In this study, PCL and collagen nanofibrous mats have been designed and characterized to meet these functional requirements. The ultimate goal of this study was to fabricate an appropriate replacement for cadaveric corneas and amniotic membranes to overcome the shortage of biological membranes for transplantation. 3D nanofibrous scaffolds with radially aligned patterns have been used in biomimetic approaches to replicate the corneal tissue structure^18,19^. In the conventional electrospinning method, a 2D nanofibrous scaffold can be produced, but it has limitations in producing a 3D nanofibrous scaffold suitable for the tissue having a curvature such as that of an eyeball (Fig. 1). The development of an electrospinning system capable of producing customizable patterned nanofibrous 3D scaffolds for hemispherical ocular tissue reconstruction remains a challenge^20^. In this experiment, 3D hemispherical nanofibrous mats with radially patterned nanofibers were fabricated using a modified rotating collector. The surface of the scaffold radially patterned with nanofibers induced cell alignment. Pore sizes of 0.5–8.0 μm were introduced to the PCL/collagen nanofibrous mat to promote the interlayer diffusion of nutrients and to promote cell-cell interactions^21–23^. The 3D nanofibrous scaffolds with radially aligned patterns were analyzed to determine the mechanical properties, transparency, contact angle, and nanotopography using field emission scanning electron microscopy (FE-SEM) and Fourier transform infrared spectroscopy (FT-IR) to determine the chemical and physical characteristics. The cell attachment, proliferation, and specific gene marker expression for rabbit corneal cells (rCCs) were determined by FE-SEM, Cell Counting Kit-8 (CCK-8) assay, and immunofluorescence. The 3D membrane was examined to verify its transparency after transplantation, and was easily handled due to the surrounding random nanofibers for transplantation into the eyes. Combined with its ability to support corneal cell function, this optical transparency and surface pattern features of the nanofiber mat enable this new biomaterial system to provide significant potential benefits for corneal tissue regeneration^24–26^. These results indicate that the 3D nanofibrous scaffolds with radially aligned patterns could be suitable substitutes for corneal grafts for ocular tissue reconstruction.

## Materials and Methods

### Materials

PCL pellets and rat tail collagen purchased from Sigma-Aldrich were used to fabricate 3D radially patterned nanofibers. 5 g of PCL pellets was stirred with a solvent of 45 g 1,1,1,3,3,3-Hexafluoro-2-propanol (HFIP) to prepare a 10 wt% PCL solution. Two hours prior to electrospinning, rat tail collagen (5 ml) was dissolved in PCL solution (20 ml) and used for electrospinning. Copper wire, a metal pin, and a hemispherical nonconductor used for the modification of the rotating collector were purchased from CosmoTech and a 20-gauge syringe needle and 12 ml syringe required for electrospinning were purchased from NORM-JECT®.

### Fabrication of a 3D radially patterned nanofibrous scaffold

**Figure 2:**
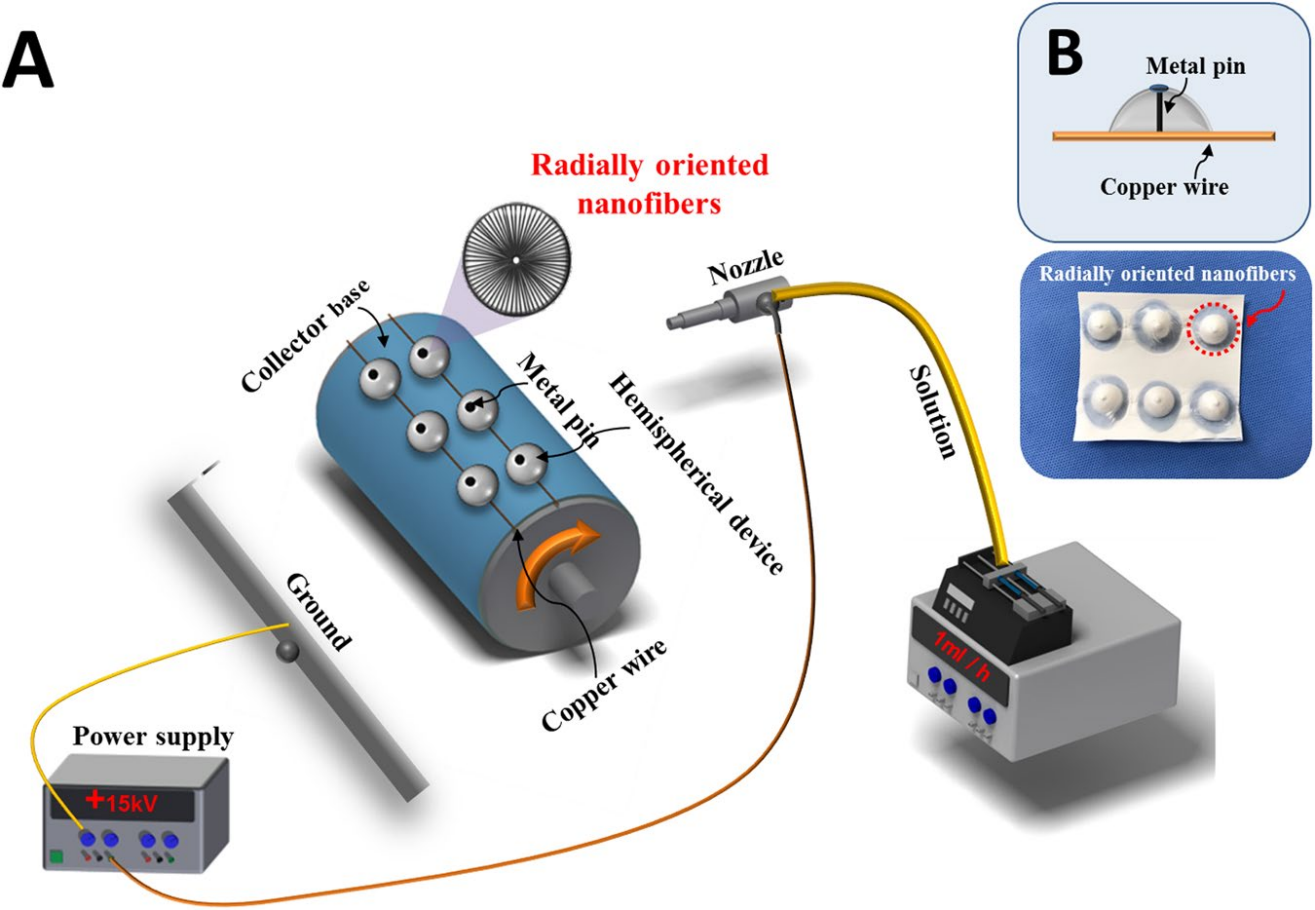
(**A**) Electrospinning setup of the fabrication of 3D radially oriented nanofibrous scaffolds, (**B**) copper wires, and metal pin in the hemispherical device designed to be electrically connected to each other.

PCL/collagen solution for the electrospinning process was prepared and the solution was magnetically stirred in a 10 ml vial for 12 hours. The prepared polymer solution was electrospun at an applied voltage of 15 kV, a tip-collector distance of 15 cm, and a solution feed rate of 1 ml/h at room temperature (25 °C). Using simple and inexpensive equipment such as copper wire and pin, we obtained a 3D radially patterned nanofibrous scaffold by inducing the change of an electric field between a rotating collector and a needle, as shown in Fig. 2. First, a rotating collector was wrapped with a polyethylene sheet with copper wires at intervals of 10 cm, and hemispherical nonconductor devices with a metal pin at the center were then attached to the copper wires. The copper wires serve to supply electricity to the metal pins in hemispherical insulated conductors. The modified collector was rotated at 1000 rpm while the electrospinning was performed, and the electrospinning was performed for a total of 10 hours. The collected nanofiber mat was dried in a vacuum oven at 40 °C for one day.

### Electric field analysis using COMSOL®

**Figure 3:**
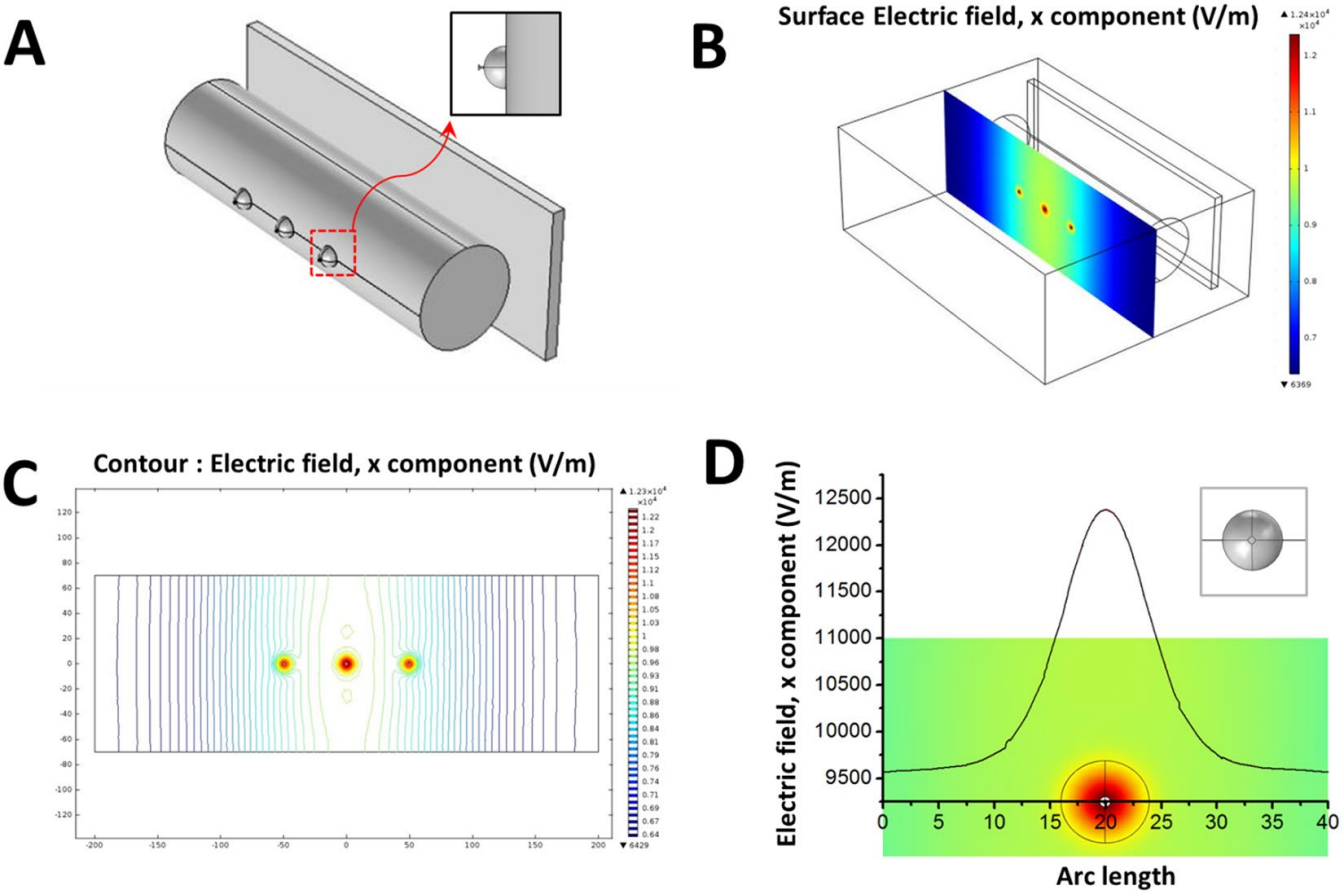
(**A**) Setup of the fabrication of the 3D nanofibrous scaffolds with radially aligned patterns (**B**) result of the electric field distribution simulation (front view): surface of electric field (**C**) contour (**D**) distance variation of electric field intensity (front view): horizontal line.

The simulation of the electric field measurement of this novel electrospinning set-up was carried out using the COMSOL® Ver.4.3 added to an AC/DC module under the Window Vista operational system. The simulation of this electrospinning method was performed using the actual configuration as shown in Fig. 3. COMSOL® enables analysis of the electric field and yields the results of the electric field distribution simulation from the front or top view.

### Fiber alignment analysis via FFT and Image J

The alignment of the nanofibers was obtained using a fast Fourier transform (FFT) method and the images were further analyzed using J software^27,28^. The fiber alignment angle of the presented SEM image was converted to an output image with grayscale pixels with nanotopographical patterns^29^. Since nanofibers are radially patterned, we divided the representative SEM images and confirmed the alignment of the nanofibers in the divided images using the FFT method. The FFT analysis data of the compartmentalized image showed the alignment of the radially patterned nanofibers.

### Characterization

The morphology of the electrospun nanofibers of the PCL/collagen nanofibrous mat was observed using SEM (Hitachi S-7400, Hitachi, Japan) and FE-SEM (Hitachi S-7400, Hitachi, Japan). Transparency was evaluated by performing the analysis spectra using a SYNERGY Mx spectrophotometer (BioTekR, USA) in the wavelength range of from 400 nm to 800 nm. The presence of rat tail collagen in the PCL nanofibers was determined by FT-IR spectroscopy (ABB Bomen MB100 spectrometer, Bomen, Canada) and the mechanical properties of the samples were determined using a universal tester (AG-5000G, Shimadzu, Japan) at room temperature. To determine the wettability of the 3D nanofibrous scaffolds with radially aligned patterns, the water contact angle of the samples was measured using a contact angle meter (GBX, Digidrop, France) with deionized (DI) water. The experiment was conducted at a room condition and at different time intervals of 5, 10, and 15 s for a total of 8 times per sample.

### Biological assessment

#### Isolation of rCCs and culture

All experiment procedures were carried out with the approval of Chonbuk National University Animal Care Committee, Jeonju, South Korea. All experiments were performed in accordance with relevant guidelines and regulations. Two female New Zealand white rabbits weighing 450 g were used in this experiment. The rabbits were anesthetized intramuscularly using 5cc of a mixture of Dormitor (1 mg/kg, Orion, Finland) and Alfaxalone (4 mg/kg, Jurox, Australia) for each rabbit. The eyes were extracted and the surrounding tissue was removed and washed in phosphate-buffered saline (PBS). The rabbit eye was washed three times with PBS and the cornea was peeled from the rabbit cornea. Collagenase A (0.2%, Roche, Germany) was used for digesting the cornea with Descemet’s membrane (DM) in an incubator at 37 °C for 1 h and the digested solution with the medium was centrifuged at 1500 rpm for 5 min. rCCs were suspended in medium containing epidermal growth factor, vascular endothelial growth factor, fibroblast growth factor (Clonetics, United States), hydrocortisone, gentamicin, amphotericin-B, and 10% fetal bovine serum (FBS).

#### Cell morphology and viability

A cell culture experiment could be the first step for testing the biocompatibility of natural or modified materials^30,31^. Isolated corneal epithelial cells, endothelial cells, corneal keratocytes, and limbal stem cells have been used as a testing protocol in ophthalmological applications^12^. In this experiment, 3D nanofibrous PCL and PCL/collagen mats with specific nano-patterns were studied as potential matrices for ocular tissue reconstruction. Their attachment and biocompatibility properties were investigated using the isolated rCCs. In addition, the impact of nanofiber orientation on the cell migration and growth were investigated. The PCL and PCL/collagen electrospun mats were cut into squares (12 mm in diameter) and sterilized for one day under UV irradiation. A 12 mm diameter cell culture plate (SPL Bioscience, Korea) in 48 wells was wrapped in a sterilized nanofibrous mat. The corneal cells isolated from the rabbit eyes were seeded on PCL and PCL/collagen nanofibrous mats at a density of 2 × 10^4^ cells per well in the culture medium of 400 μl of Dulbecco Modified Eagle Medium (DMEM). Cells were inoculated in DMEM high glucose medium supplemented with 10% FBS and 1% penicillin/streptomycin at 37 °C in a humidified atmosphere of 5% CO_2_. Fresh medium was added every 2 days to all samples. After culturing for 1, 3, and 5 days, the cell morphology was observed using SEM and a confocal laser scanning microscope (LSM 510 META, Carl Zeiss, Germany). The biocompatibility was then confirmed by the CCK assay. The CCK solution (60 μl) was added to each well and cultured at 37 °C for 2 hours in an incubator. In each sample, a mixed solution of 100 μl of the cell culture medium and CCK solution was added to a 96-well plate, and the absorbance was confirmed at 450 nm using a microplate reader (Tecan, Austria). The results were presented as the mean ± standard error of the mean.

#### Migration test

The samples (random and radially aligned fibrous membranes) were cut into squares (12 mm in diameter) and sterilized for 24 hours under UV irradiation. A 12 mm diameter cell culture plate was wrapped in a sterilized electrospun fibrous membrane. The rCCs were seeded on PCL and PCL/collagen fibrous membranes at a density of 2 × 10^4^ cells per well in the culture medium of 400 μl of DMEM high glucose medium supplemented with 10% FBS and 1% penicillin/streptomycin at 37 °C. After culturing for 3 days, the scratch test was adapted to electrospun membranes with a 5 × 3 × 3mm^3^ stainless steel strip for cell migration studies along their surface. Confocal laser-scanning microscopy (LSM 510 META, Carl Zeiss, Germany) were used for observing cell migration after culture for 3, 4, 5, 6, and 8 days.

#### DNA quantification assay

The measurement of cellular growth in terms of DNA content was determined using a Quant-iT^TM^ PicoGreen^TM^ dsDNA Assay Kit (Life Technologies, USA). The DNA assay is based on the measurement of the fluorescence of Picogreen which is a nucleic acid strain for quantitating double-stranded DNA (dsDNA) in solution. The 10 μl of lysate was mixed with 190 μl of Pico Green in TE buffer (1 mM EDTA, 10 mM Tris-HCl, pH 7.5) and incubated for 5 min at room temperature, protected from light. Fluorescence was measured with a SYNERGY Mx spectrophotometer (BioTekR, USA) in black 96-well cell culture plates with excitation wavelength at 480 nm and emission wavelength at 520 nm. The samples with cells were harvested after 1, 3, and 5 days to evaluate the construct cellularity, that was assessed by determining the DNA content. The amount of cell DNA on each sample was expressed in ng/cm^2^ and the assay was repeated using a dilution of the sample to confirm the quantitation results.

#### *In-vitro* Immunohistochemical examinations

For *in-vitro* immunohistochemical analysis, the cultured rCCs were fixed in 4% paraformaldehyde in PBS for 10 min at room temperature and the samples were incubated for 10 min with PBS containing 0.1% Triton X-100. 1 M Quenching solution was added to the samples for 15 min for permeabilization of the cells. After washing the rCCs in PBS several times for 5 min, a protein blocking solution (DAKO) was added for 12 min in a dark room at room temperature. After nonspecific blocking, all samples were incubated with anti-zona ocludin-1 (ZO-1) (1:100, Santa Crux Biotechnology) as a primary antibody for 90 min at room temperature. Alexa Fluor 594-conjugated AffiniPure Donkey Anti-mouse IgG (1:250, Santa Crux Biotechnology, USA) was used for ZO-1 detection. Finally, all samples were mounted with mounting medium with 4′,6-diamidino-2-phenylindole (DAPI) (Santa Crux Biotechnology, USA) and immunofluorescence images were obtained using a confocal LSM.

#### mRNA Expression

Total ribonucleic acid (RNA) was extracted from rCCs cultured for 1, 3, and 5 days on control group(TCP), random, and radially aligned fibrous membranes. The rCCs cultured on the samples were washed with PBS and treated with the total RNA isolation solution (RiboEx^TM^, GeneAll, Korea). Extracted RNA samples were quantified using an Eppendorf BioSpectrometer (Eppendorf, Germany). Expression of mRNAs of the samples was confirmed by related genes such as ZO-1, Na+/K+-ATPase (NaK), and chloride channel protein 3 (CLCN3). Every samples were denatured for 30 s at 95 °C and elongated for 1 min per 1 kb at 72 °C. Products of the PCR were separated by electrophoresis at 100 V on a 0.7% agarose gel (Lonza, Korea) in a 0.5% TAE buffer (Showa Chemical, Korea) and visualized using ethidium bromide (Sigma-Aldrich, Korea).

### Statistical analysis

Data are presented as a mean ± standard error of the mean and analyzed by one-way ANOVA. A p < 0.05 was taken as statistically significant.

## Results and Discussion

### Electric field analysis

The electric fields are created by electric charges or varying magnetic fields. Polymeric fibers are fabricated by a charged polymer jet oriented at external electric fields in an electrospinning process. Highly aligned or customizable patterned nanofibers and nanofibrous mats can be produced by manipulating electric fields^28,29^. In this study, the nanofibers were collected using a hemispherical 3D device with a metal pin and were then radially ordered because of the adjustment of the electric fields between the needle tip and the rotating collector. The electric field simulation of the novel electrospinning was carried out using COMSOL® Ver. 4.3, as shown in Fig. 3. The simulation was performed using the actual configuration of this electrospinning method as presented in Fig. 3A. The COMSOL® program enables analysis of the electric fields from the front view, the representative distribution of the electric fields on the surface, and from a contour whereby the direction is denoted by a colored scale bar as shown in Fig. 3B and C. In addition, Fig. 3D shows the effect of the distance variation of electric field intensity (front view) and the figure is analyzed from the horizontal line in order to quantitatively analyze the results. Due to the metal pin in the hemispherical 3d shaped conductor, the electric field is most prominent in the center of the scaffold, which helps the nanofibers collect in a radially aligned orientation. The electric field on the metal pin is represented in red in Fig. 3B–D. Due to the difference in the electric field between the metal pin and the circular periphery, the nanofibers can be stretched and aligned, which enables the 3D nanofibrous scaffolds to be fabricated with radially aligned patterns^20^. These results clearly demonstrate that the electric field is distributed and a successful 3D nanofibrous mat with radially aligned patterns is obtained.

### Characterization of 3D radially oriented nanofibrous scaffold

Figure 2 shows the setup for the fabrication of a 3D radially oriented nanofibrous scaffold. The nanotopography of the nanofibers can be controlled by simply changing the electrospinning setup such as the applied voltage, solution flow rate, and modification of the collector^28,29,32^. In this experiment, due to the modified rotating collector, the electric field between the needle tip and the collector was changed, thereby making it possible to manufacture 3D radially oriented nanofibers. Copper wires and pins in the hemispherical device attached to the rotating collector are designed to be electrically connected to each other, as shown in Fig. 2B.

**Figure 4:**
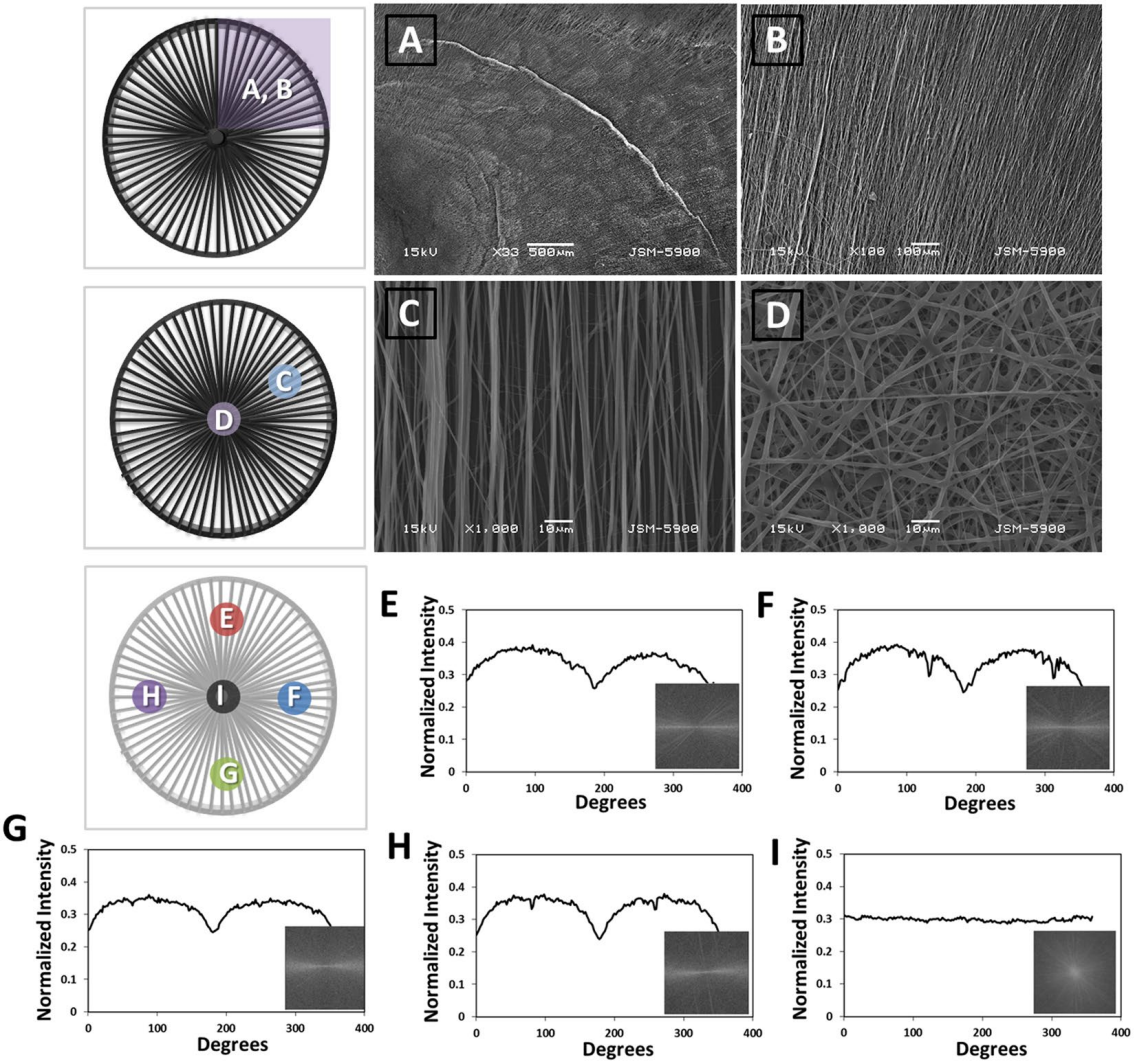
(**A**,**B**) SEM images of radially oriented aligned nanofibers on the (**A**) and (**B**) parts shown in the illustration. (**C**) SEM image of aligned nanofibers on the C part shown in the illustration. (**D**) SEM image of random nanofibers on the D part shown in the illustration. (**E**–**I**) FFT output images for the nanofibers with pixel intensity plots against the angle of acquisition of each part of the letter, shown on the 3D radially aligned nanofibrous mat.

SEM images of the electrospun scaffold fabricated in this electrospinning setup are shown in Fig. 3A–D. It was found that the nanofibers fabricated on the hemispherical equipment are radially arranged, except for the portion of the metal pin (Fig. 4C). On the other hand, random nanofibers were collected on the metal pin (Fig. 4D). Alignment analysis of the nanofibers by FFT reveals that radially and circumferentially aligned fibers were successfully produced as presented in Figs. 4E–I. The radially aligned nanofibers produce ordered gray pixels; however, the randomly oriented nanofibers produce symmetrically distributed pixels in the output image. The shape of the peaks in the plot also reflects the degree of alignment of the nanofibers in the sample. As the degree of alignment of the nanofibers in the sample increased, the peaks became sharper and more pronounced compared to those in the graph of the random nanofibers. Radially-oriented nanofibers were produced by an electric field formed using an outer, non-conductive hemispherical device and a metal center pin. Unlike a conventional electrospinning setup for the fabrication of a 2D nanofibrous mat, the electrospinning method is capable of producing a hemispherical scaffold such as a contact lens and is suitable for application to a curved tissue such as that of the eye, elbow, or 3D wound site. In addition, conventional 3D electrospinning has a limitation whereby it is difficult to pattern the internal nanofibers^33^.

**Figure 5:**
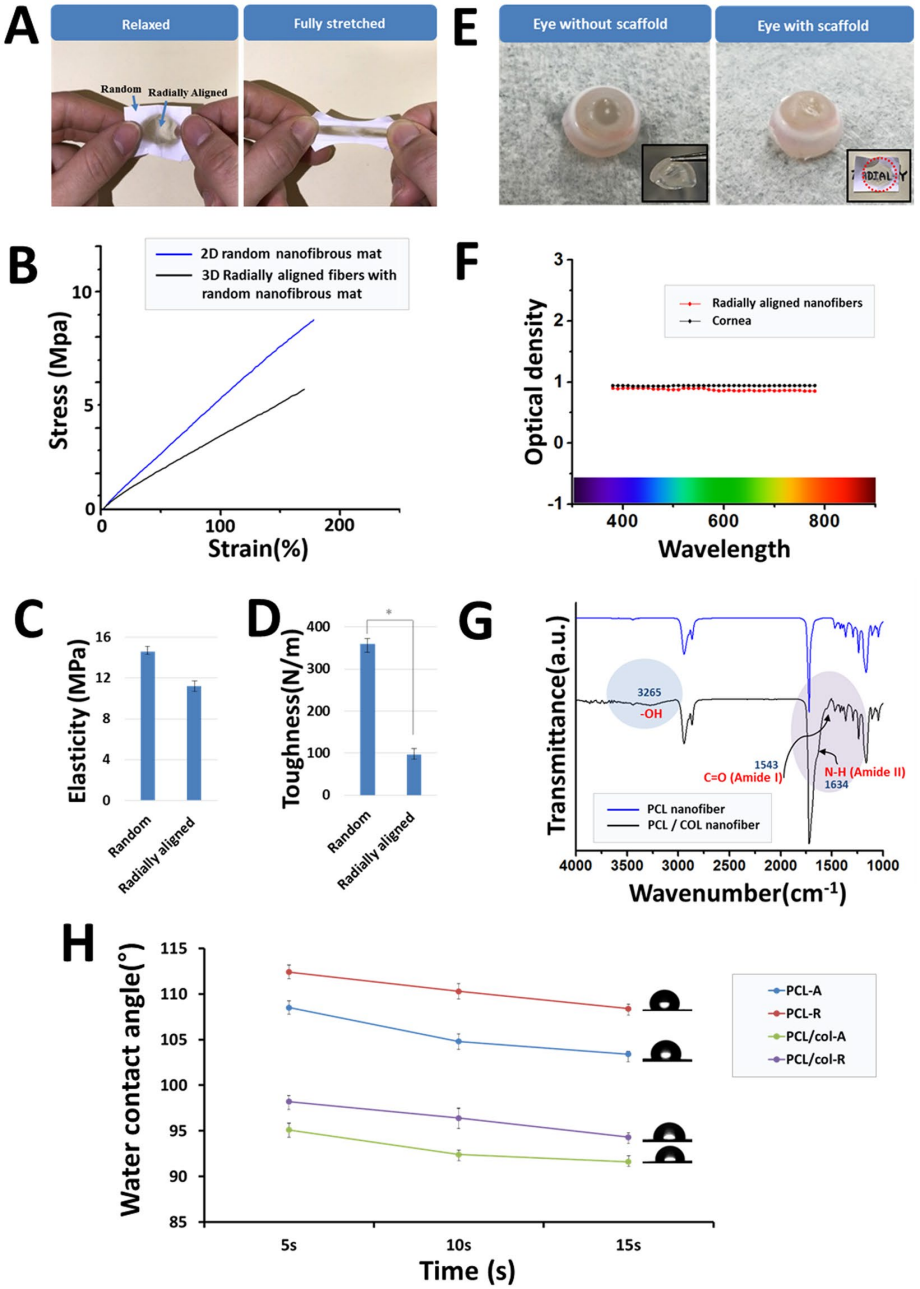
(**A**) Radially aligned nanofibrous mat with relaxed (left) and fully stretched (right). (**B**) Representative stress-strain curves of the radially aligned PCL/collagen mats. (**C**,**D**) Toughness and elasticity of PCL/collagen mats (radially aligned and randomly oriented nanofibrous mats), showing the single mat of a radially aligned nanofibrous mat in the form of a 3D hemisphere. (**E**) Digital photographs of an eye without the scaffold and an eye with the scaffold (inset: digital photographs of cornea and a mat. The letters confirm the transparency of the radially aligned nanofibrous mat). (**F**) Transparency results using spectrum analysis method in the wavelength range of 400 nm–800 nm. (**G**) FT-IR spectra of the PCL and PCL/collagen nanofibrous mats. (**H**) Water contact angle plot of the PCL and PCL/collagen nanofibrous mats.

The relaxed and fully stretched nanofibrous scaffold, as shown in Fig. 5A, confirmed that the fabricated 3D PCL/collagen nanofibros mat has sufficient mechanical strength as a corneal or wound dressing scaffold. The mechanical testing was carried out using a universal testing machine at room temperature condition and the speed head testing was set at 5 mm/min. Figure 5B shows the stress-strain curves of the 2D random nanofibrous mat and 3D radially aligned nanofibers with a random nanofibrous mat under tensile loading. Two samples showed a comparatively linear region with a slope in the initial stress-strain curve. The toughness and elasticity of the mats significantly differed, as shown in Fig. 5C and D. The toughness of the 2D randomly oriented nanofibrous mat and the 3D radially aligned nanofibrous mats was 372 ± 30 N/m and 97 ± 20 N/m, respectively. The elasticity of the 2D randomly oriented nanofibrous mat and 3D radially aligned nanofibrous mat were 13 ± 1 MPa and 11 ± 1 MPa, respectively. The 2D randomly oriented nanofibrous mat showed a higher mechanical strength relative to the 3D radially aligned nanofibrous mat. An electrospun membrane consisting of aligned or radially aligned nanofibers has lower mechanical strength because of their fewer contact points between the fibers than an electrospun fibrous mat made of randomly oriented nanofibers. A lower mechanical properties were observed and reported in the aligned or radially aligned fibrous constructs regardless of the kinds of polymer. It is challenging not only to fabricate a neat membrane or hemispherical 3D form with aligned nanofibers but also to use this for surgical applications as a corneal scaffold. Also, the corneal scaffold should have transparency, but if the aligned or radially aligned nanofibers are thickened to increase the mechanical strength, the transparency will be lost. For this reason, it is important to develop a method of manufacturing a corneal scaffold having both adequate mechanical strength and transparency. The radially aligned 3D scaffold developed through this study has a higher mechanical strength than the previously developed aligned nanofibrous scaffold and native cornea tissue, since the periphery of the 3D radially aligned scaffold is connected to a membrane made of random nanofibers. The mechanical property of the acellular pig cornea after incubating in PBS for 1 h has studied by Kong *et al*. The proposed 3D radially aligned scaffold can also be seen that the mechanical strength is good enough to be used as a corneal scaffold when compared to the physical properties of the native corneal tissue.

Transparency is an important factor for a cornea scaffold, not only for monitoring the process of healing but also for the recovery of the patient’s vision after implantation^34–36^. The transparency of the radially oriented PCL nanofibrous mats blended with collagen was analyzed using a spectrophotometer in the wavelength range from 400 to 800 nm. The optical intensity of the cornea was about 0.1 in the wavelength range from 400 to 800 nm. As shown in Fig. 5F, the prepared scaffold was similar to the transparency of the cornea when wetted with PBS solution. Thus, the transparency of the radially oriented nanofibrous scaffolds has an acceptable value for the cornea scaffold for implantation. In addition, transparency was visually confirmed through photographs of the eye and the eye implanted with scaffolds, as shown in Fig. 5E. The scaffold also has good transparency, even when seen with the naked eye.

FT-IR spectroscopy was used to analyze the presence of collagen in the electrospun PCL nanofibers and the change in the chemical structure of the collagen. The graph in Fig. 5G shows a band of 3000–3300 cm^−1^ indicating the stretching of the hydroxyl group and NH groups of the collagen groups. In addition, a band was identified at 1634 cm^−1^, typical of the vibration mode of amide I groups and the third band at 1543 cm^−1^ is attributed to the amide II due to collagen immobilization. The results of the PCL/collagen peak intensity indicated that collagen was uniformly distributed throughout the PCL nanofibers, as shown in Fig. 5G.

Cell attachment to the scaffold, which is essential for cell migration, proliferation, and differentiation, can be greatly affected by the chemical composition or the physical surface architecture of the scaffolds. The measurement of the contact angle can be an important assessment for the evaluation of cell attachment when the cells are seeded on the scaffolds. The contact angle between the scaffold and the substrate was measured at 5, 10, and 15 s in the experiment. The time dependency of the water contact angle of the scaffold can influence the initial stage of the cell attachment after cell seeding because the seeded cells can adhere more rapidly on the scaffold where it absorbs the cell adhesion molecules (CAM)^29^. The contact angles of the randomly oriented and radially aligned PCL/collagen nanofibrous mat were measured in the experiment; the average contact angles after 10 seconds were 97.5 and 94.7, respectively. The measurements showed higher contact angles for the PCL nanofibrous mat. The increase of the contact angle could be attributed to the increased porosity or the shape of the pores of the nanofibers. This result shows that the aligned nano-patterned surface modification of the scaffolds can be advantageous for the production of scaffolds with high contact angles^37,38^.

### Cell proliferation

**Figure 6:**
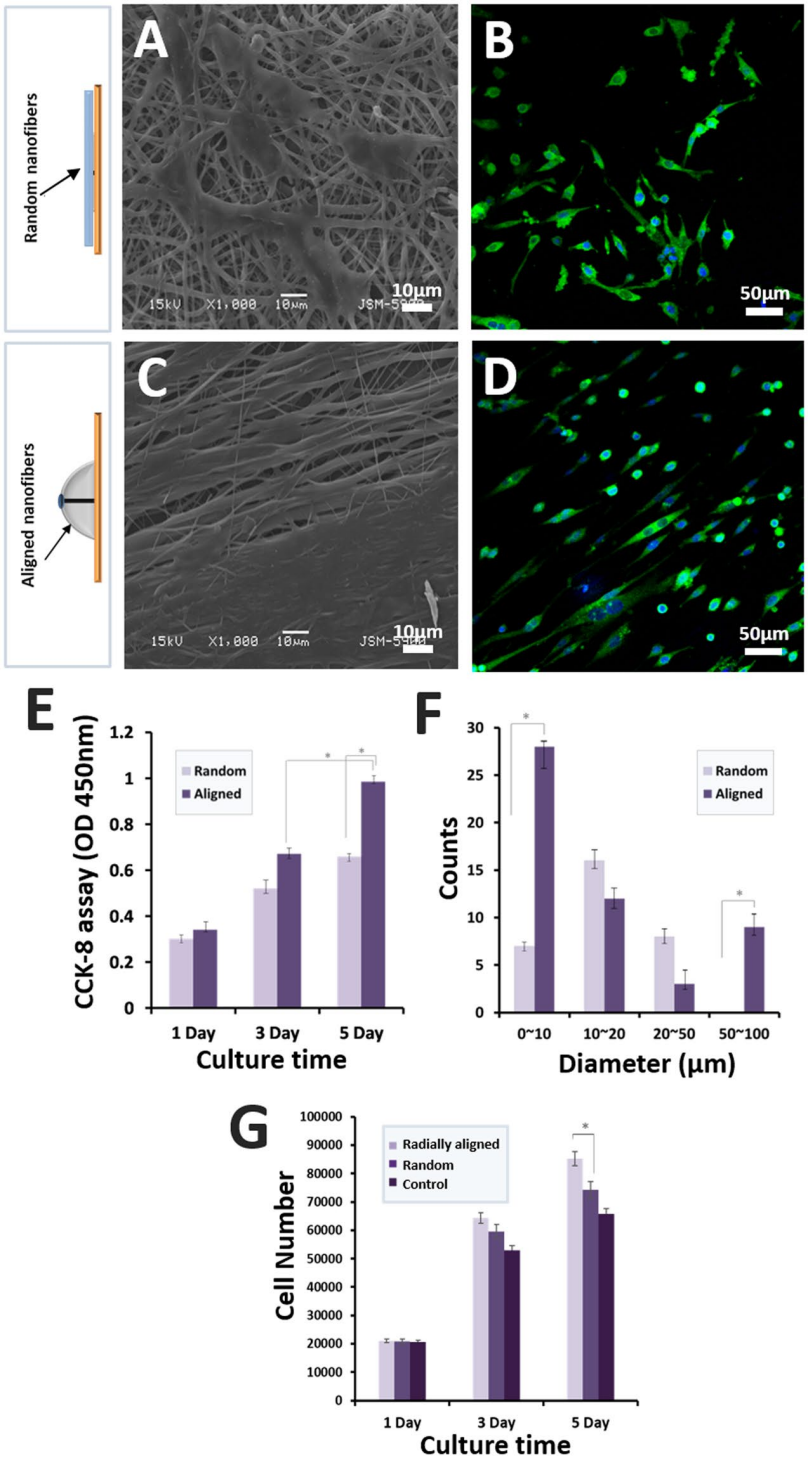
(**A**) SEM image of corneal cells of a rabbit attached after 3 days of culture on a randomly oriented nanofibrous mat. (**B**) Confocal microscopy images of corneal cells of a rabbit attached after 3 days of culture on a randomly oriented and aligned nanofibrous mat. (**C**) SEM image of corneal cells of a rabbit attached after 3 days of culture on an aligned nanofibrous mat. (**D**) Confocal microscopy images of corneal cells of a rabbit attached after 3 days of culture on an aligned nanofibrous mat. Actin Green 488 (green) was applied for actin filament and DAPI (blue) for staining nuclei. (**E**) CCK-8 assay result of corneal cells of rabbit on random and radially aligned nanofibrous mats after 1, 3, and 5 days of cell culture. (**F**) Distribution of cell size for corneal cells of a rabbit attached after 3 days of culture on randomly oriented and aligned nanofibrous mats. (**G**) Pico Green dsDNA quantification assay.

The topographical characteristics of the corneal scaffolds are crucial for cell interaction and the proliferation rate of the corneal cells^39^. The FE-SEM images in Fig. 4 show the surface topography of the samples without cell seeding. The scaffolds constituted of aligned nanofibers mimicking the native ECM by providing a 3D nano-pattern to induce cell growth or tissue regeneration. According to previous studies, aligned or radially oriented scaffolds provide significant potential benefits for corneal regeneration and *in situ* correction of the corneal stroma. The morphology of corneal cells on the samples was evaluated by FE-SEM and confocal images after being cultured for 3 days (Fig. 6A–D). Compared to the randomly oriented nanofibrous sample, the FE-SEM image of the radially aligned nanofibrous sample shows a better cell proliferation rate. The confocal image also shows a higher cell proliferation rate in radially aligned nanofibrous scaffolds, further confirming the orientation of the rCCs. The proliferation of corneal cells on the matrixes for transplantation is important for the recovery of vision^36,39^. The proliferation of corneal cells in a different topography of the nanofibrous scaffolds was evaluated using CCK-8 assay after 1, 3, and 5 days of cell seeding (Fig. 6E). No remarkable difference is observed between the rates of cell proliferation among the experimental groups on day 1. However, the radially aligned nanofibrous sample shows a significant increase compared to the randomly oriented nanofibrous sample after 3 days of cell culture. Proliferation results of the FE-SEM, confocal images, and CCK-8 assay suggest that the orientation of nanofibers in the fabricated scaffold could play an important role in the behavior of corneal cells or anisotropic tissue. The 3D radially oriented nanofibrous scaffolds showed excellent ability to induce cell morphogenesis and cell migration as well as mechanical properties. The degree of alignment of the nanofibers also affected the size of the rCCs. On the randomly oriented nanofibrous mat, rCCs with the size of 10–20 um were the most common, and on the aligned nanofibrous mat, the spherically shaped rCCs smaller than 10 um were the most common (Fig. 6F). On the aligned nanofibrous mat, however, elongated cells larger than 50 um and smaller than 100 um were observed. These results appear to be due to the difference in pore size and alignment among the nanofibrous mats.

The amount of cell DNA on the radially aligned nanofibrous 3D mats was higher than that on the randomly oriented nanofibrous mats (Fig. 6G). The amount of cell DNA on the control polystyrene dishes was significantly lower than that on the proposed hemispherical 3D nanofibrous scaffolds with radially aligned patterns.

### rCCs migration

The cells are surrounded by the ECM, the complex network consisting of molecules, proteins, and polysaccharides. The cells in the native tissues migrate in response to various gradients of stimulation^13,40^. The regulation of the cell migration is of paramount importance in biomedical application field^41^. One effective way is a creation of a microenvironment which mimics the target tissue complexity by incorporating biological and physical gradients into a scaffold. Topographical cues like porosity, pore size, alignment, and stiffness of the scaffold can be crucial for cell migration and behavior, inducing cell polarity and controlling the cellular activities and proliferation rate^40^. The proposed hemispherical 3D nanofibrous scaffolds with radially aligned patterns in the manuscript not only have aligned topographic features like aligned nanofibrous mat but also have gradients of stimuli such as porosity and pore size that can affect the polarity and migration rate of the cells. The constructs developed in this study have the advantages (including a transparency) of both ordered nanofibers and gradient materials. This is also shown in other papers about the fabrication of the radially oriented fibrous constructs. Xie *et al*. have demonstrated the method of manufacturing an electrospun fibrous structure consisting of radially aligned PCL fibers to mimic the dura mater and to enhance the movement of the cells from the surrounding tissue to the center of dural defects^42^. Their scaffolds based on radially aligned fibers showed great potential as dural scaffolds to induce wound healing and regeneration.

**Figure 7:**
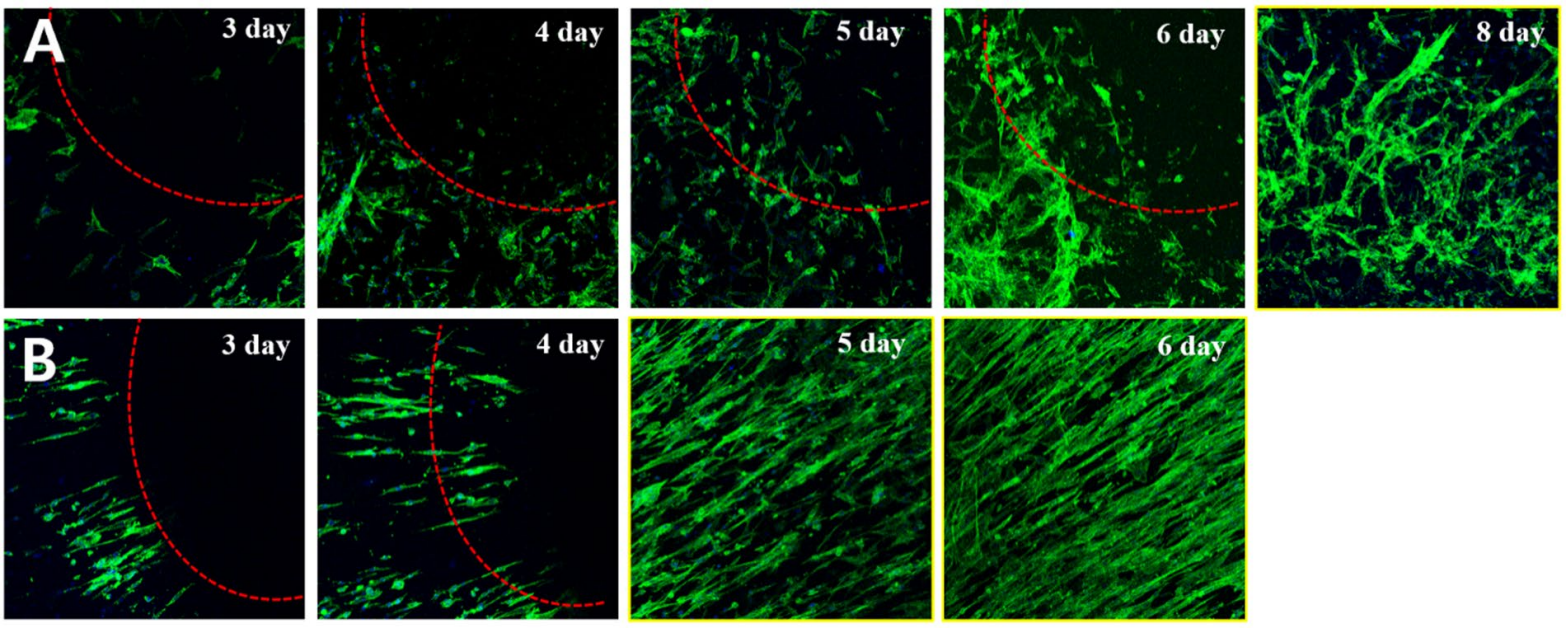
(**A**,**B**) Fluorescence images comparing the cell migration when rCCs were cultured on the membranes of randomly oriented and radially aligned fibers, respectively, for 8 days. The scratch test adapted to the mats with a 5 × 3 × 3mm^3^ stainless steel strip for cell migration studies along their surface topography.

To evaluate cell migration and motility on this membrane, rCCs were stained with actin green and fluorescence images were taken at different times. The scratch test was adapted to the membranes with a stainless steel strip along their surface on day 3 after seeding. Figure 7 showed rCCs distribution after seeding on the scaffolds of random and radially aligned fibers on day 3, 4, 5, 6, and 8 day. The ability for rCCs to repopulate the simulated defect was measured for cell migration assay. The void area decreased with increasing culture time for the membranes because of the inward migration of rCCs. The hemispherical 3D nanofibrous scaffolds with radially aligned patterns can significantly promote migration of cells when compared to randomly oriented 2D fibrous mats. Even after 6 day of scratching on the random fibrous membrane, the rCCs showed a residual bare surface of about 4mm^2^ as shown in Fig. 7A. However, the cells migrated and recovered the entire portion of the defect after 5 day of culture time for the radially aligned fibrous membrane (Fig. 7B).

### Immunohistochemical analysis

**Figure 8:**
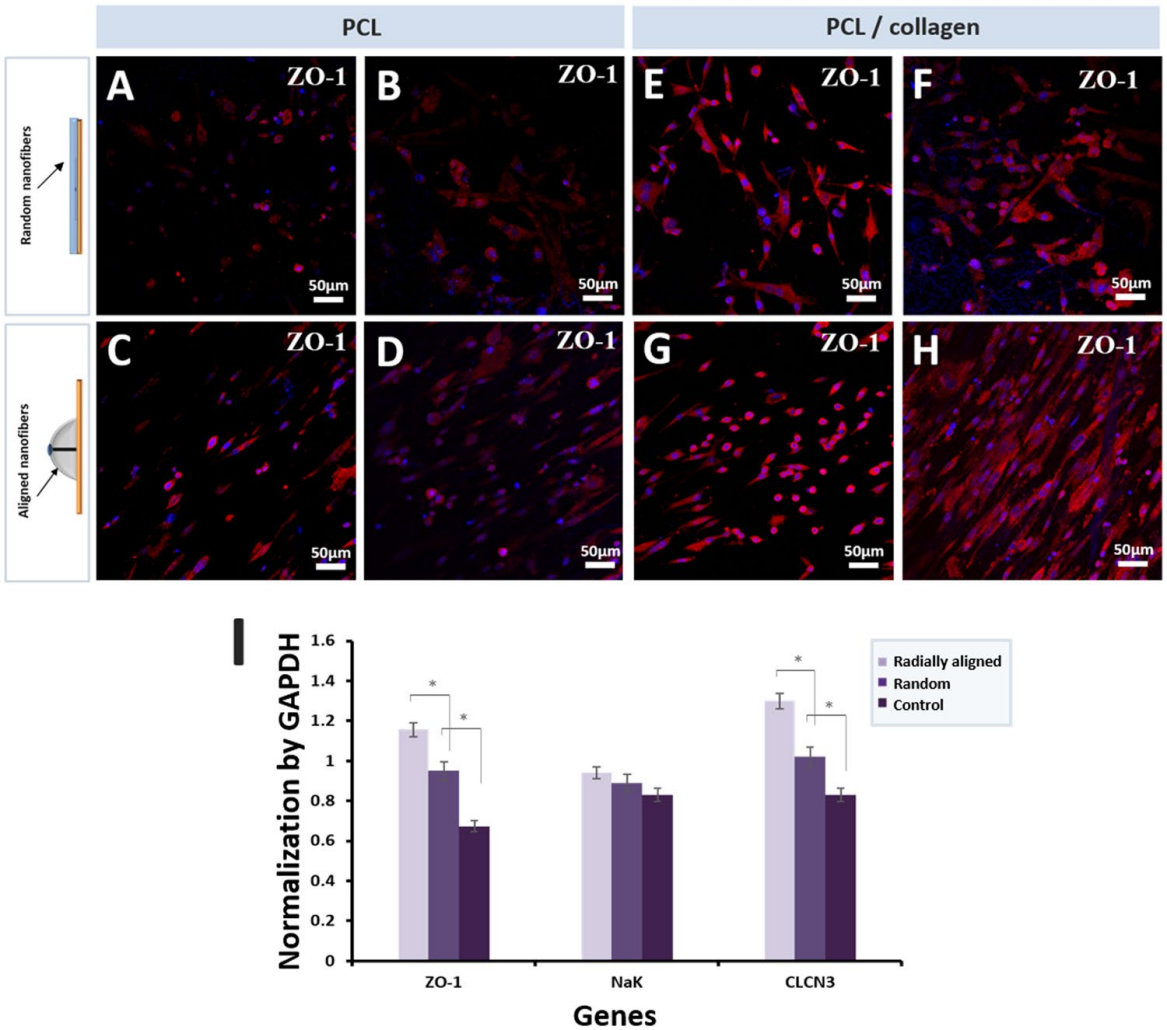
(**A**,**B**) Immunofluorescent staining of ZO-1 in the corneal cells after 3 and 7 days culture on the PCL random nanofibrous scaffolds. (**C**,**D**) Immunofluorescent staining of ZO-1 in the corneal cells after the 3 and 7 days culture on the PCL aligned nanofibrous scaffolds. (**E**,**F**) Immunofluorescent staining of ZO-1 in the corneal cells after the 3 and 7 days culture on the random PCL/collagen nanofibrous scaffolds. (**G**,**H**) Immunofluorescent staining of ZO-1 in the corneal cells after the 3 and 7 days culture on the aligned PCL/collagen nanofibrous scaffolds (DAPI stained nuclei of the corneal cells were used for normalization). (**I**) Specific gene expression of rCCs by RT-PCR (normalized by GAPDH).

Immunohistochemical analysis results with ZO-1 staining of the scaffolds with rCCs demonstrate that the rCCs and the matrixes were attached with their retained function as shown in Fig. 8. The relative quantity of zona ocludin-1 (ZO-1) in the corneal cells is important to the properties of the mats for cornea regeneration^43^ because ZO-1 determines the distribution of F-action and functions as a major cytoskeletal organizer in endothelial cells^44,45^. The expression of ZO-1 of the rCCs on the radially aligned nanofibrous scaffolds shows a much higher intensity of staining and denser stains than on the randomly oriented nanofibrous scaffolds. Moreover, the cultured rCCs on the ordered nanofibrous matrixes show the better organization of ZO-1.

### mRNA Expression

To confirm the expression of m RNA, gene markers for rCCs such as ZO-1, Na+/K+-ATPase (NaK), and CLCN3 were used as shown in Fig. 8I. All gene markers were normalized by Glyceraldehyde-3-phosphate dehydrogenase (GAPDH). CLCN3 that plays the role of regulator of pH, proliferation, and immigration of cell-to-cell is important in keeping the proper size and morphology of rCCs. NaK that is an enzyme found in cell membrane facilitates maintenance of the transparency of cornea through control of the edema of pump function. Compared with that of the randomly oriented fibrous membrane, most of the genes showed a higher expression rate in the radially oriented fibrous membrane. The rCCs on the hemispherical 3D nanofibrous scaffolds with radially aligned patterns showed highly enhanced expression of ZO-1 and CLCN3. Compared to the control group (TCP), electrospun fibrous membranes have favorable environments for typical gene expression of rCCs, especially 3D nanofibrous scaffolds with radially aligned patterns, and provide for prominent roles in cell proliferation and adhesion. As a result, the 3D nanofibrous scaffolds with radially aligned patterns show great potential as artificial corneal substrates and may open novel avenues for clinical carriers with maintaining the phenotype of rCCs.

## Conclusions

We designed a new electrospinning setup to enable the production of a hemispherical 3D nanofibrous scaffold consisting of radially aligned nanofibers that mimic the isotropic tissues that grow in a directional orientation. Unlike current electrospinning methods, the developed electrospinning method is capable of producing hemispherical scaffolds such as contact lenses, and is designed for application to curved tissues such as those of the eye, elbow, or 3D wound area. These results demonstrate the considerable potential of 3D scaffolds for tissue that requires transparency and hemispherical design. Moreover, the ordered patterns on the scaffolds enable the regulation of the rCCs proliferation rate. The aligned topography of the fabricated matrixes provides favorable environments for critical functions of cultured rCCs. We expect this 3D nanofibrous scaffold can also be used as a corneal therapeutic platform with other techniques such as surface treatment, biomolecules conjugation, drug-delivery system, etc.
