# Author Correction: Fabrication of transparent hemispherical 3D nanofibrous scaffolds with radially aligned patterns via a novel electrospinning method

**DOI:** 10.1038/s41598-018-24842-w

**Published:** 2018-04-24

**Authors:** Jeong In Kim, Ju Yeon Kim, Chan Hee Park

**Affiliations:** 1 Department of Bionanosystem Engineering, Graduate School, Chonbuk National University, Jeonju, 561–756 Republic of Korea; 2 Division of Mechanical Design Engineering, College of Engineering, Chonbuk National University, Jeonju, 561-756 Republic of Korea

Correction to: *Scientific Reports* 10.1038/s41598-018-21618-0, published online 21 February 2018

In Figure 7B, the ‘4 day’ slide is incorrect. The correct Figure 7 appears below as Figure 1.

**Figure 1:**
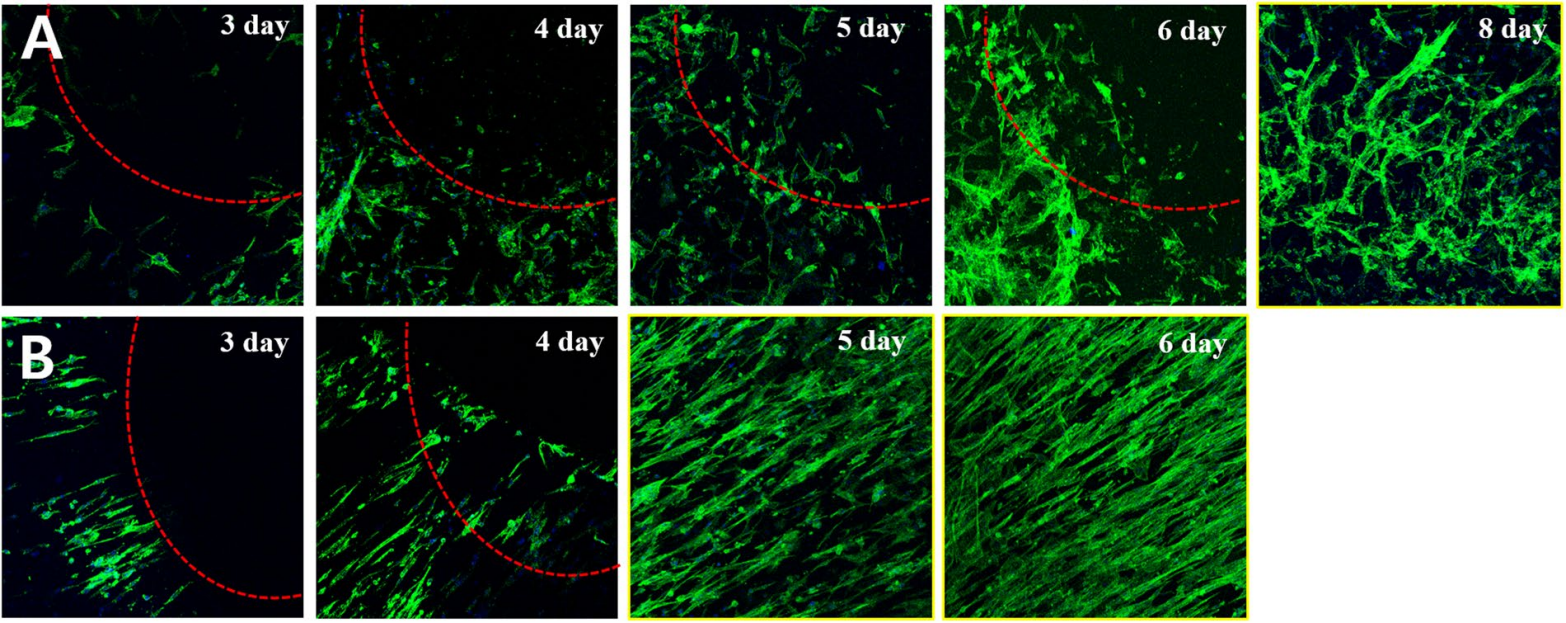
(**A**,**B**) Fluorescence images comparing the cell migration when rCCs were cultured on the membranes of randomly oriented and radially aligned fibers, respectively, for 8 days. The scratch test adapted to the mats with a 5 × 3 × 3 mm^3^ stainless steel strip for cell migration studies along their surface topography.

